# The hnRNP A2B1 is important for the replication of SFTSV and other RNA viruses

**DOI:** 10.1128/spectrum.00829-24

**Published:** 2024-08-21

**Authors:** Xu Zhang, Li-na Yan, Bin-yan Liu, Chuan-min Zhou, Xue-jie Yu

**Affiliations:** 1 State Key Laboratory of Virology, School of Public Health, Wuhan University, Wuhan, China; 2 Department of Infectious Diseases, Tangdu Hospital, Air Force Medical University, Xi’an, China; 3 Center for Environment and Health in Water Source Area of South-to-North Water Diversion, School of Public Health, Hubei University of Medicine, Shiyan, China; 4 The First Hospital of Hebei Medical University, Shijiazhuang, China; Shandong First Medical University, Jinan, Shandong, China

**Keywords:** SFTSV, hnRNP A2B1, RNA virus, nucleoprotein

## Abstract

The heterogeneous nuclear ribonucleoprotein (hnRNP A2B1) is a key component of the hnRNP complex involving RNA modulation in eukaryotic cells and it has also been reported to be involved in the replication of the hepatitis E virus, influenza A virus, and hepatitis B virus. However, it is not clear whether the role of the hnRNP A2B1 in viral replication is conserved among RNA viruses and what is the mechanism of hnRNP A2B1 in RNA virus replication. In this study, we first used severe fever with thrombocytopenia syndrome virus (SFTSV), a tick-borne RNA virus that causes a severe viral hemorrhagic fever as well as other RNA viruses including VSV-GFP, SeV, EV71, and ZIKV to demonstrate that knockout hnRNPA2B1 gene inhibited viral RNA replication and overexpression of hnRNP A2B1 could restore the RNA levels of all tested RNA viruses. These results suggest that hnRNPA2B1 upregulation of viral replication is conserved among RNA viruses. Next, we demonstrated that hnRNP A2B1 was translocated from the nucleus to the cytoplasm under RNA virus infection including SFTSV, VSV-GFP, SeV, EV71, and ZIKV, suggesting translocation of hnRNP A2B1 from the nucleus to the cytoplasm is crucial for RNA virus replication. We then used SFTSV as a model to demonstrate the mechanism of hnRNP A2B1 in the promotion of RNA virus replication. We found that overexpression of SFTSV nucleoprotein can also cause hnRNP A2B1 translocation from the nucleus to the cytoplasm and that the SFTSV NP interacted with the RNA recognition motif 1 domain of hnRNP A2B1. We further demonstrated that the hnRNP A2B1 interacted with the 5′ UTR of SFTSV RNA. In conclusion, we revealed that the hnRNP A2B1 upregulation of viral RNA replication is conserved among RNA viruses; the mechanism of hnRNP A2B1 in promotion of SFTSV viral RNA replication is that SFTSV NP interacted with the hnRNPA2B1 to retain it in the cytoplasm where the hnRNP A2B1 interacted with the 5′ UTR of SFTSV RNA to promote the viral RNA replication.

**IMPORTANCE:**

Severe fever with thrombocytopenia syndrome virus (SFTSV) is a tick-borne RNA virus with a high mortality rate of up to 30%. In this study, we first used SFTSV as a model to demonstrate that the role of hnRNPA2B1 in viral replication is conserved in SFTSV. Then we used other RNA viruses, including VSV-GFP, SeV, EV71, and ZIKV, to repeat the experiment and demonstrated the same results as SFTSV in all tested RNA viruses. By knocking out the hnRNPA2B1 gene, SFTSV RNA replication was inhibited, and overexpression of hnRNPA2B1 restored RNA levels of SFTSV and other tested RNA viruses. We revealed a novel mechanism where the SFTSV nucleoprotein interacts with hnRNPA2B1, retaining it in the cytoplasm. This interaction promotes viral RNA replication by binding to the 5′ UTR of SFTSV RNA. The findings suggest that targeting hnRNPA2B1 could be a potential strategy for developing broad-spectrum antiviral therapies, given its conserved role across different RNA viruses. This research provides significant insights into the replication mechanisms of RNA viruses and highlights potential targets for antiviral interventions.

## INTRODUCTION

Severe fever with thrombocytopenia syndrome virus (SFTSV) is a tick-borne bunyavirus that causes a severe hemorrhagic fever termed severe fever with thrombocytopenia syndrome (SFTS). SFTS is an emerging infectious disease that was first reported in China in 2011 and then in East Asia and Southeast Asian countries (1–4). The major clinical manifestations of SFTS include fevers, encephalitis, meningitis and multiple organ failure, thrombocytopenia, and leukopenia with a high case fatality of up to 30% (1). Despite the clinical significance, specific therapeutics against SFTSV are unavailable due to the lack of knowledge on host immunity, especially the cellular factor that contributes to SFTSV replication. As the host-vector range expands, there is an increased risk of transmission and the number of countries reporting infections is increasing.

The SFTSV is an RNA virus and its genome consists of three negative-strand RNA segments (5). The L segment encoding viral RNA-dependent RNA polymerase (RdRp) is a key player in virus transcription and genome replication, with a size of 6,368 nucleotides, which contains multiple domains and functions. During the processes of genome replication and transcription, L protein synthesizes three distinct RNA species: antigenomic complementary RNA (cRNA), genomic viral RNA (vRNA), and capped, mostly non-polyadenylated viral mRNA. Genome replication is believed to be initiated *de novo* by the L protein, while viral transcription is dependent on short, capped RNA primers derived from cellular RNAs by a mechanism called cap-snatching (6–8). M segment encodes glycoprotein precursors, and S segment encodes nucleoprotein (NP) and nonstructural proteins (NSs). NP is essential for the transcription and replication of viral life cycles and genomes, while NSs play an essential role in SFTSV propagation and can form viroplasm-like structures (VLSs) in infected and transfected cells (9–11). Typically, the SFTSV life cycle is divided into four steps, namely internalization, replication, assembly, and exocytosis (12). Briefly, following the interaction between viral glycoproteins and cell factor, SFTSV is transported into the cytoplasm *via* endosomes. The acidic environment in late endosomes induces a conformational change in SFTSV glycoproteins and then triggers the release of genomic RNA into the cytoplasm for replication. The SFTSV genome is encapsulated by NP and RdRp to form an RNP complex. Assembled bunyavirus RNPs are incorporated into Golgi or ERGIC-originated phagophores by interacting with glycoprotein and then released into the extracellular space *via* autophagic secretory vesicles (8, 13, 14).

Heterogeneous nuclear ribonucleoproteins (hnRNPs) are RNA-binding proteins with a conserved structure involved in gene transcription, post-transcriptional modification, and maturation of precursor mRNAs (15). The hnRNP protein family consists of 20 proteins, hnRNPs can modulate virus replication by directly interacting with viral RNA or protein components or indirectly *via* regulating host gene expression (16).

The hnRNP A2B1 is a key component of the hnRNP complex in mammalian cells, known for controlling RNA splicing (17). It also mediates cellular signal transduction and is expressed in various cells, including lung, liver, breast, pancreatic, and glioma (18). It regulates pathopoiesis through multiple mechanisms and is involved in the immune response and inflammation progression, particularly during viral infection (19). The roles of hnRNPA2B1 in virus infection are complex, as it exerts pro-inflammatory, anti-inflammatory, or pathopoiesis effects through interactions with multiple DNAs, RNAs, and proteins. The hnRNP A2B1 partnership is affected by its expression, localization, and post-translational modifications (17, 20). As a result, it has become the therapeutic target of many viral infectious diseases. The latest knowledge about the biological roles of hnRNPA2B1 in viral infections is essential for understanding the intersection between viral RNA or protein and hnRNP A2B1 (21). Recent studies suggest that hnRNP A2B1 has a wide range of post-translational modifications regulating its activity and responding to cellular stress processes through changes in subcellular localization (17). However, no detailed mechanism has been determined for hnRNP A2B1 mediating multiple RNA functions through binding to specific sequences *in vivo* (22). The latest research determined the crystal structures of the tandem RRM domain of hnRNP A2B1 in complexes with various RNA substrates, elucidating the specific recognition of ARG and UAG motifs by the RNA recognition motif 1 (RRM1) and RRM2 domains (23). HnRNP A2B1 is a crucial protein in innate immunity, particularly during viral infection (24, 25). A previous study suggests that hnRNP A2B1 promotes the replication of RNA viruses, such as influenza A virus ribonucleoprotein (vRNP), which can interact and colocalize with nuclear protein (NP), knockdown of hnRNP A2B1 decreases viral RNA synthesis, suggesting it may act as a positive regulator of influenza A viral RNP activity (17). In 2019, hnRNP A2B1 was identified as a novel nuclear DNA sensor, which recognizes viral DNA to initiate the activation of IFN signaling and inhibits DNA virus HSV-1 replication (26).

However, no interaction or functional study has been reported with cellular hnRNP A2B1 for SFTSV replication. Previous studies have demonstrated hnRNPs could interact with the virus proteins of influenza A virus, enterovirus 71, hepatitis C virus (HCV), and hepatitis E virus for replication (17, 27–29). Since SFTSV is also a member of a RNA virus and SFTSV NP protein is an RNA-binding protein, we hypothesized that SFTSV NP protein might also be able to interact with hnRNP A2B1 during SFTSV infection. Therefore, we determine whether hnRNP A2B1 plays a key role in the life cycle of SFTSV in this study.

## RESULTS

### HnRNP A2B1 is upregulated and translocated under SFTSV infection

**Fig 1:**
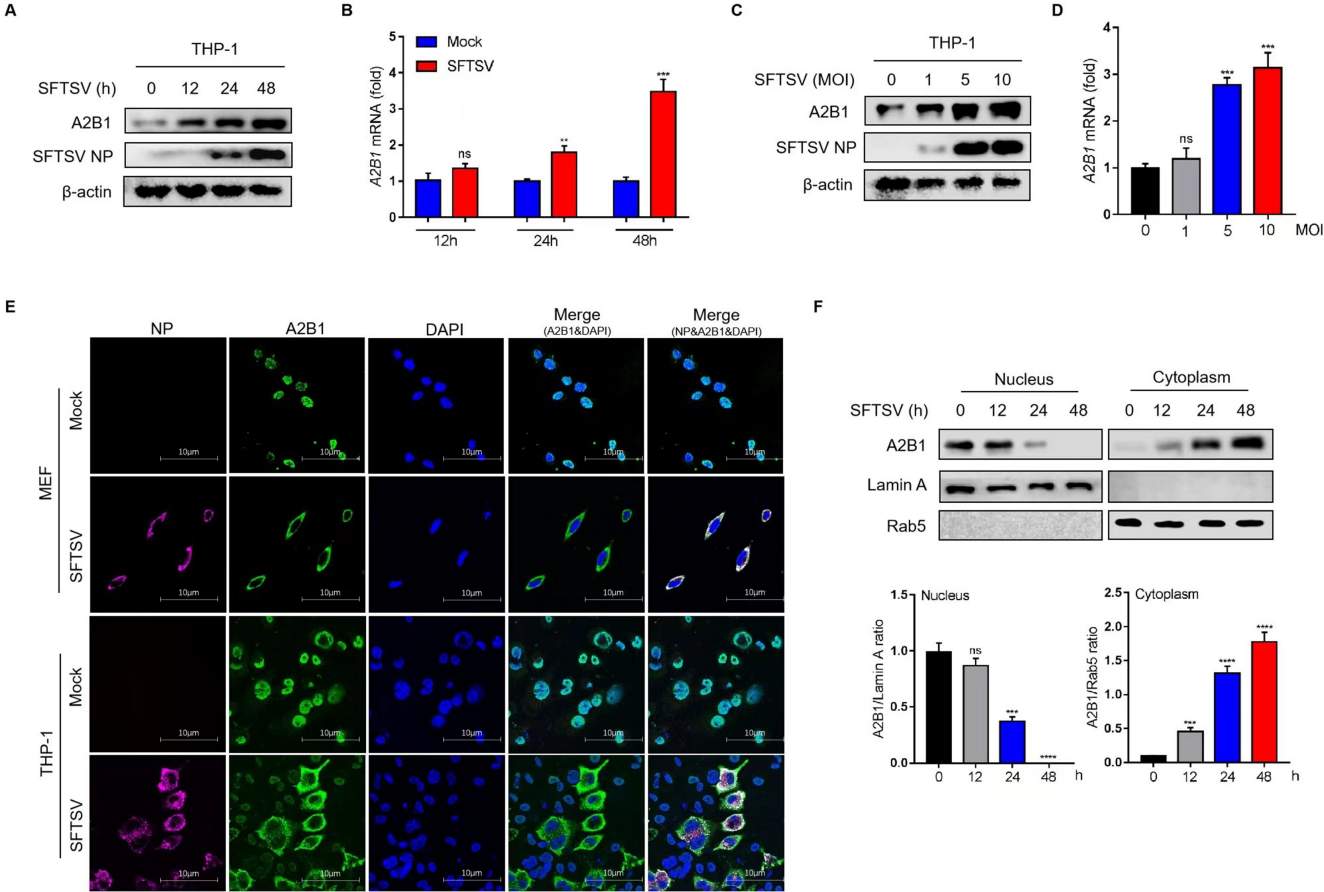
HnRNP A2B1 is upregulated and translocated under SFTSV infection. (**A**) THP-1 cells were infected with SFTSV at a multiplicity of infection (MOI) of 10 for 12, 24, or 48 h. A2B1 protein levels were analyzed with Western blot. (**B**) THP-1 cells were infected with SFTSV at an MOI of 10 for 12, 24, or 48 h. *A2B1* mRNA levels were analyzed with RT-qPCR. (**C**) THP-1 cells were infected with SFTSV at an MOI of 0, 1, 5, or 10 for 48 h. A2B1 protein levels were analyzed with Western blot. (**D**) THP-1 cells were infected with SFTSV at an MOI of 0, 1, 5, or 10 for 48 h. *A2B1* mRNA levels were analyzed with RT-qPCR. (**E**) MEF cells and THP-1 cells were infected with SFTSV at an MOI of 10 or 0 (Mock control) for 48 h, SFTSV NP (purple), hnRNP A2B1 (green), and DAPI (blue) were analyzed with confocal microscopy. (**F**) MEF cells were infected with SFTSV at an MOI of 10 for the indicated time points. Nuclear and cytoplasmic proteins were separated. hnRNP A2B1, Lamin A, and Rab5 protein levels were analyzed with Western blot. Lamin A and Rab5 were nuclear and cytoplasmic index proteins, respectively. Nuclear and cytoplasmic Western blot data were semi-quantified and normalized against Lamin A and Rab5 protein loading control, respectively. Data were obtained from three independent experiments (*n* = 3). **P < 0.01, ***P < 0.001, ****P < 0.0001, ns, not significant.

In our study, to identify whether hnRNP A2B1 was involved in the infection of RNA virus SFTSV, the protein and mRNA levels of hnRNP A2B1 were analyzed in SFTSV-infected human macrophage cell line, THP-1. The results indicated that the expression and transcription of hnRNP A2B1 were significantly increased under SFTSV infection in a time and dose-dependent manner (Fig. 1). We then detected the subcellular distribution of hnRNP A2B1 under SFTSV infection with confocal microscopy. Interestingly, hnRNP A2B1 was mainly distributed in the nucleus in a steady state, while it was located in the cytoplasm in SFTSV-infected mouse embryonic fibroblast (MEF) and THP-1 cells (Fig. 1E). To further confirm the translocation of hnRNP A2B1, nuclear and cytoplasmic distributions of hnRNP A2B1 proteins were analyzed in SFTSV-infected cells with Western blot. Consistent with confocal microscopy results, immunoblotting data showed that the hnRNP A2B1 gradually accumulated in the cytoplasm and almost disappeared in the nucleus at 48 h after SFTSV infection (Fig. 1F). These results indicated that hnRNP A2B1 is upregulated with SFTSV infection and it is translocated from the nucleus to the cytoplasm, suggesting hnRNP A2B1 is involved in the SFTSV infection.

### HnRNP A2B1 upregulates SFTSV replication

**Fig 2:**
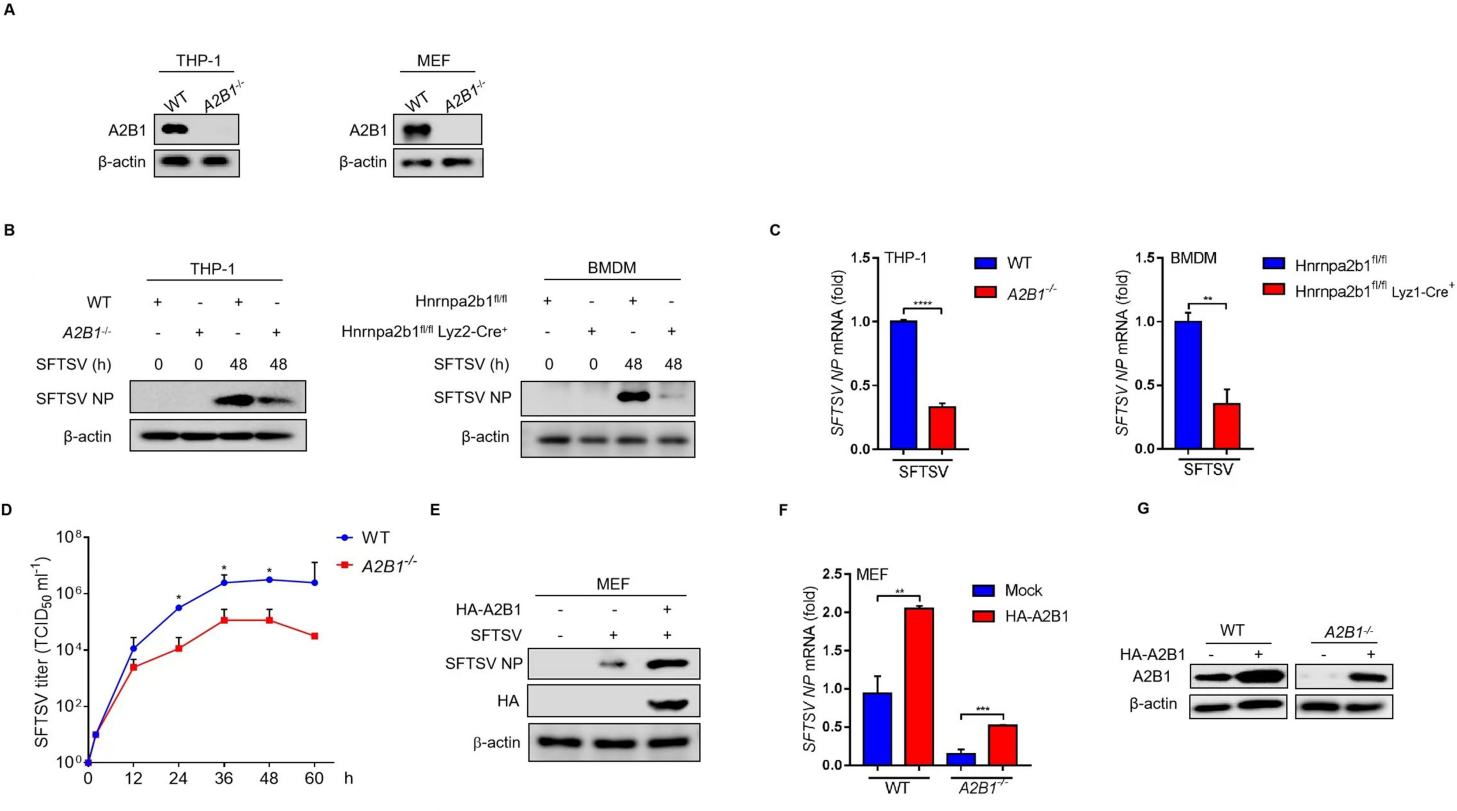
HnRNP A2B1 upregulates SFTSV replication. (**A**) Knockout of hnRNP A2B1 in THP-1 cells and MEF cells was confirmed with Western blot. (**B**) BMDM cells were isolated from *A2B1*^fl/fl^ and *A2B1*^fl/fl^*Lyz2-Cre^–/–^* mice. WT and *A2B1*^-/-^ THP-1 and BMDM cells were infected with SFTSV at an MOI of 10 for 48 h, and SFTSV NP protein levels were analyzed with Western blot. (**C**) WT and *A2B1*^-/-^ THP-1 and BMDM cells were infected with SFTSV at an MOI of 10 for 48 h, and *SFTSV NP* mRNA levels were analyzed with RT-qPCR. (**D**) WT and *A2B1*^-/-^ MEF cells were infected with SFTSV for the indicated time points. SFTSV titers in cell culture supernatant were measured with immunofluorescence assay. (**E**) MEF cells were transfected with HA-tagged A2B1 for 24 h and then were infected with SFTSV at an MOI of 10 for 48 h. SFTSV NP protein levels were analyzed with Western blot. (**F**) WT and *A2B1*^-/-^ MEF cells were transfected with HA-tagged A2B1 for 24 h, and then were infected with SFTSV at an MOI of 10 for 48 h. and *SFTSV NP* mRNA levels were analyzed with RT-qPCR. (**G**) WT and *A2B1*^-/-^ MEF cells were transfected with HA-tagged A2B1 for 24 h, A2B1 protein levels were analyzed with Western blot. Data were obtained from three independent experiments (*n* = 3). **P < 0.01, ***P < 0.001, ****P < 0.0001, ns, not significant.

To determine the impact of hnRNP A2B1 on SFTSV replication, we examined the protein and mRNA levels of SFTSV NP in SFTSV-infected THP-1 and mouse bone marrow-derived macrophages (BMDM) cells. First, we constructed hnRNP A2B1 knockout THP-1 and MEF cells using the CRISPER/Cas 9 system (Fig. 2A). Immunoblotting and RT-qPCR results showed that the protein and mRNA levels of NP were impaired remarkably in *A2B1^-/-^* THP-1 and BMDM cells (Fig. 2B and C). To investigate the effect of hnRNP A2B1 on SFTSV replication, the growth curves of SFTSV in WT and *A2B1^-/-^* MEF cells were measured after virus infection. In addition, the viral titers of SFTSV in cell culture supernatant were analyzed with immunofluorescence. We found that SFTSV titers in cell culture supernatant of *A2B1^-/-^* MEF cells were much lower than that in WT cells at different time points after virus infection (Fig. 2D). Furthermore, overexpression of hnRNP A2B1 could increase the protein and mRNA levels of NP in MEF cells during SFTSV infection (Fig. 2E and F), and SFTSV replication could be restored partly in *A2B1^-/-^* MEF cells under hnRNP A2B1 overexpression status (Fig. 2F and G). These results suggest that hnRNP A2B1 upregulates SFTSV replication.

### HnRNP A2B1 upregulation viral replication is conserved among RNA viruses

**Fig 3:**
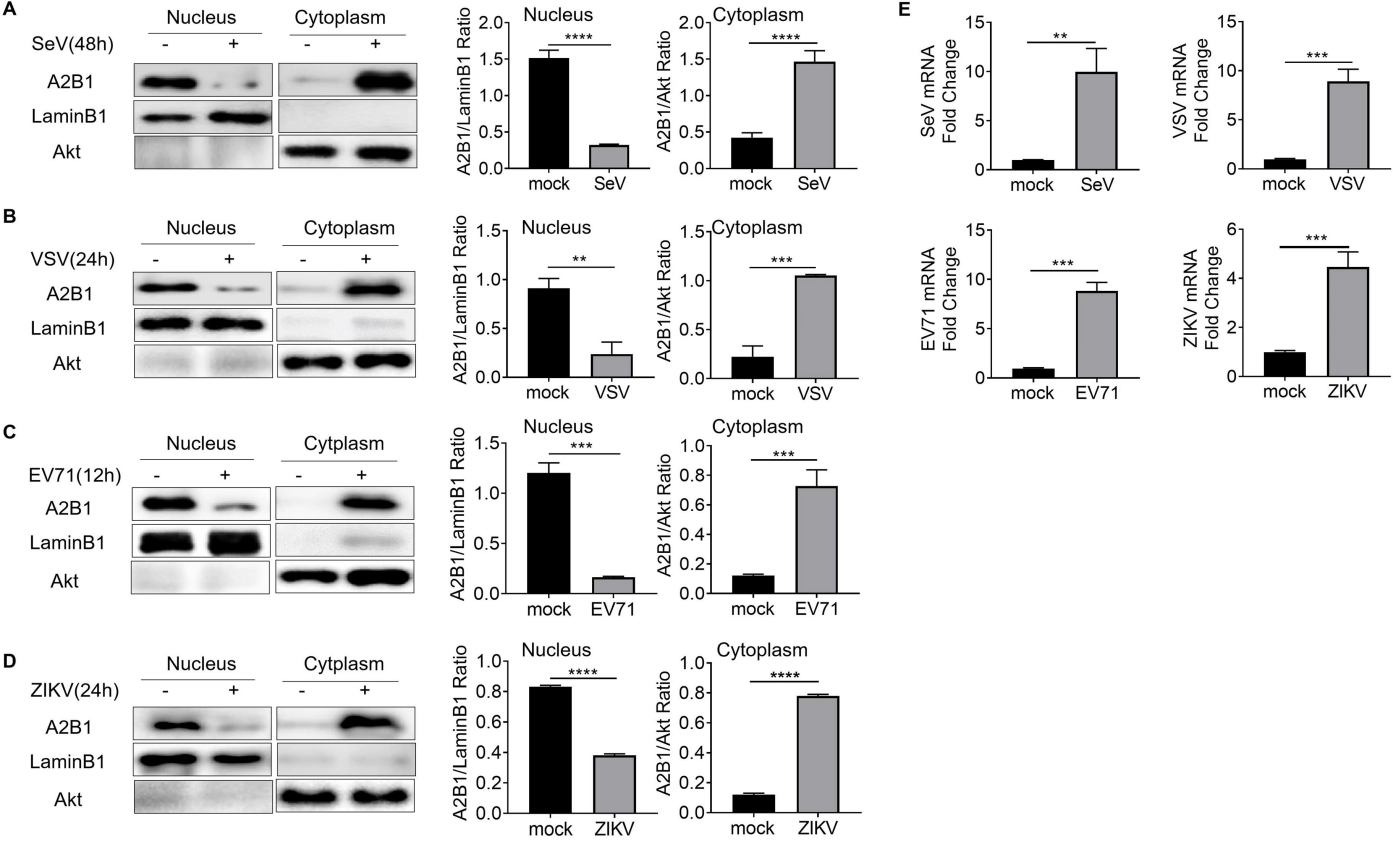
HnRNP A2B1 undergoes nucleocytoplasmic translocation after infection with other RNA viruses. (**A–D**) THP-1 cells were infected with SeV, VSV, EV71, or ZIKV at an MOI of 10 at the indicated time for each virus. Nuclear and cytoplasmic proteins were separated. A2B1, Lamin B1, and Akt protein levels were analyzed with Western blot. Lamin B1 and Akt were nuclear and cytoplasmic index proteins, respectively. Nuclear and cytoplasmic Western blot data were semi-quantified and normalized against LaminB1 and Akt protein loading control, respectively. (**E**) THP-1 cells were infected with SeV, VSV, EV71, and ZIKV at an MOI of 10 for 48 h, 24 h, 12 h, and 24 h. SeV, VSV, EV71, and ZIKV mRNA levels were analyzed with RT-qPCR, respectively.

**Fig 4:**
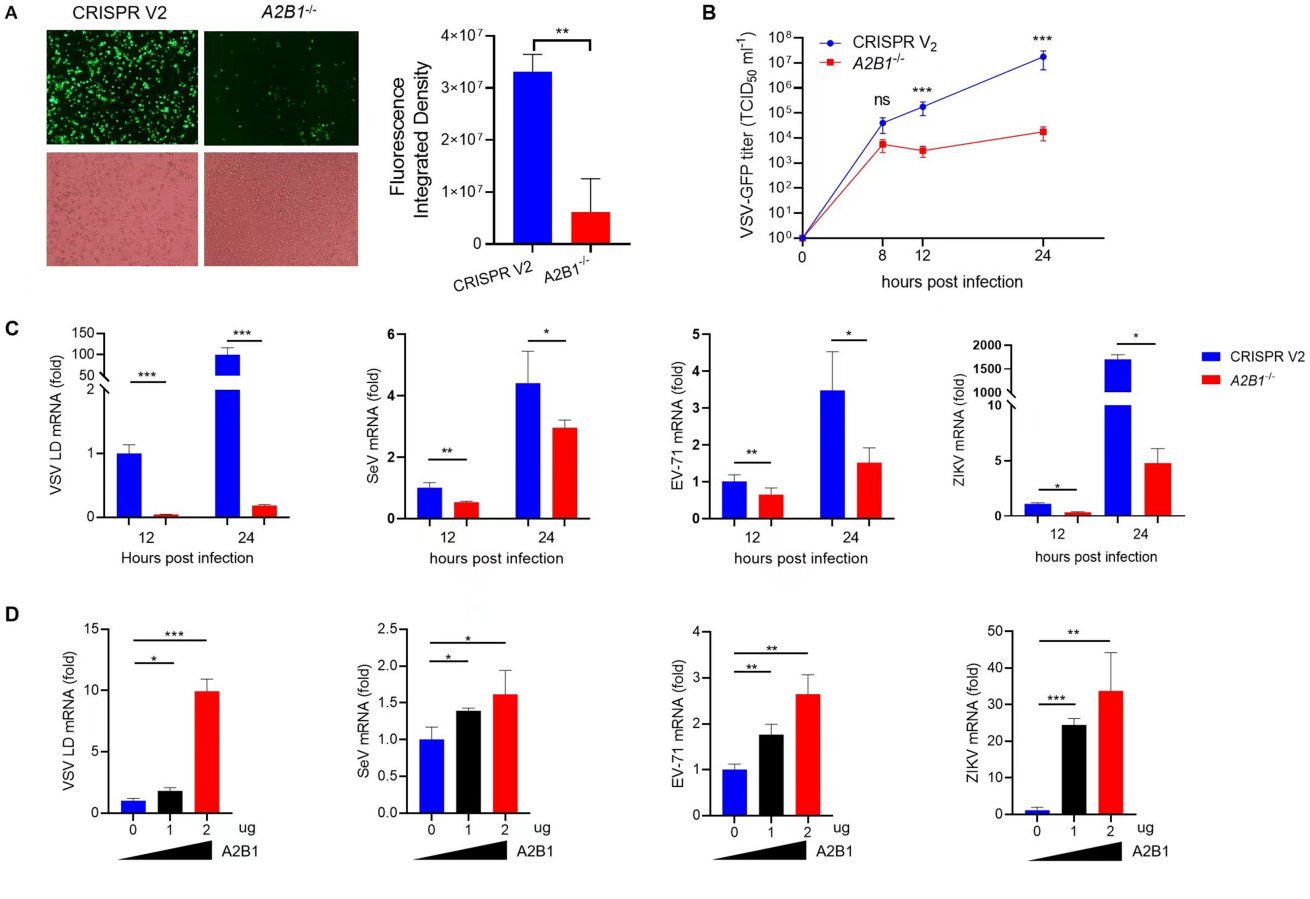
HnRNP A2B1 upregulation of viral replication is conserved among RNA viruses. (**A**) WT and *A2B1*^-/-^ THP-1 cells were infected with VSV-GFP at an MOI of 10 for 24 h. Green fluorescence was observed with a fluorescence microscope, and cells were observed with an inverted microscope (left). The relative fluorescence intensity was calculated using Image J software (light). (**B**) WT and *A2B1*^-/-^ THP-1 cells were infected with VSV-GFP virus at an MOI of 10 for the indicated time points. VSV-GFP virus titers in cell culture supernatant were measured with TCID_50_. (**C**) WT and *A2B1*^-/-^ THP-1 cells were infected at an MOI of 10 with VSV-GFP, SeV, EV71, or Zika for 12 h and 24 h, respectively. Viral mRNA levels were analyzed with RT-qPCR. (**D**) MEF cells were transfected with HA-tagged A2B1 for 24 h and then were infected at an MOI of 10 with VSV-GFP, SeV, EV71, or ZIKV for 12 h and 24 h, respectively. Viral mRNA levels were analyzed with RT-qPCR. Data were obtained from three independent experiments (*n* = 3). *P < 0.05, **P < 0.01, ***P < 0.001, ns, not significant.

To explore whether the phenomenon of viral infection-induced translocation of hnRNP A2B1 was conserved among RNA viruses. Cells were infected with RNA viruses including Sendai virus (SeV), Vesicular stomatitis virus (VSV-GFP), Enterovirus 71 (EV71), and Zika virus (ZIKV), respectively. Immunoblotting data showed that hnRNP A2B1 was translocated from the nucleus to the cytoplasm in all of these RNA viruses (Fig. 3). Wildtype THP-1 cells and *A2B1^-/-^* THP-1 were used to investigate the role of hnRNP A2B1 during the infection of these RNA viruses. At 24 h after VSV-GFP infection, the VSV-GFP can be observed in both WT and knockout THP-1 cells under a fluorescence microscope (Fig. 4A). Virus titers of VSV-GFP were reordered at 8, 12, and 24 h after infection. We found that VSV-GFP titers in the cell culture supernatant of *A2B1^-/-^* THP-1 cells were much lower than that in WT THP-1 cells (Fig. 4B). In addition, we also found that the viral mRNA levels of SeV, EV71, and ZIKV were greatly reduced in *A2B1^-/-^* THP-1 cells at 12 and 24 h after virus infection (Fig. 4C). Furthermore, overexpression of hnRNP A2B1 could increase the mRNA levels of VSV-GFP, SeV, EV71, and ZIKV (Fig. 4D). These results suggest that A2B1 upregulated the replication of the tested RNA viruses.

### SFTSV NP is important for translocation of A2B1

**Fig 5:**
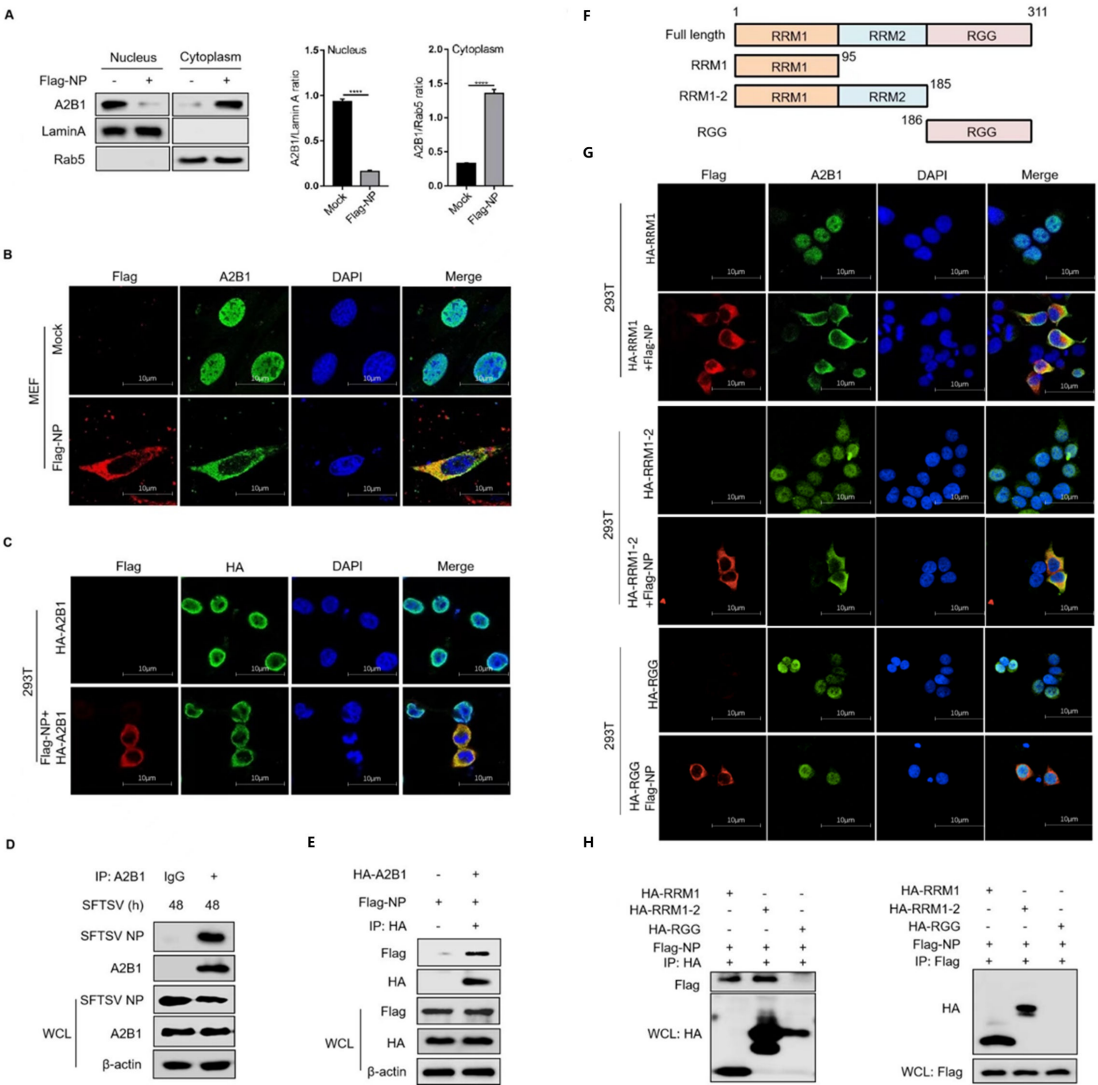
SFTSV NP is important for the translocation of hnRNP A2B1. (**A**) MEF cells were transfected with Flag-tagged SFTSV NP for 24 h, nuclear and cytoplasmic proteins were separated, and hnRNP A2B1, Lamin A, and Rab5 protein levels were analyzed with Western blot. Western blot data were semi-quantified and normalized against Lamin A and Rab5 protein loading control, respectively. (**B**) MEF cells were transfected with Flag-tagged SFTSV NP for 24 h, Flag-tagged SFTSV NP (red), A2B1 (green), and DAPI (blue) were analyzed with confocal microscopy. (**C**) HEK293T cells were transfected with the indicated plasmids for 24 h, Flag-tagged SFTSV NP (red), HA-tagged A2B1 (green), and DAPI (blue) were analyzed with confocal microscopy. (**D**) THP-1 cells were infected with SFTSV at an MOI of 10 for 48 h, and interaction between SFTSV NP and A2B1 in THP-1 cells was analyzed with CO-IP. (**E**) HEK293T cells were transfected with HA-tagged A2B1, Flag-tagged SFTSV NP for 24 h, and interaction between Flag-tagged NP and HA-tagged A2B1 was detected with CO-IP. (**F**) The diagram of hnRNP A2B1 domains and the truncated hnRNP A2B1 construction. (**G**) HEK293T cells were transfected with the indicated plasmids for 24 h, Flag-tagged SFTSV NP (red), HA-tagged hnRNP A2B1 domains (green), and DAPI (blue) were analyzed with confocal microscopy. (**H**) HEK293T cells were transfected with the indicated plasmids for 24 h, and interaction between Flag-tagged NP and HA-tagged hnRNP A2B1 domains was detected by CO-IP. Data were obtained from three independent experiments (*n* = 3). ****P < 0.0001.

Considering the colocalization between SFTSV NP and hnRNP A2B1 in the cytoplasm during SFTSV infection (Fig. 1E), we hypothesized that the nucleocytoplasmic translocation of hnRNP A2B1 could be mediated by SFTSV NP. To verify our hypothesis, MEF cells were overexpressed with SFTSV NP. Interestingly, immunoblotting results showed that overexpression of SFTSV NP could promote the translocation of hnRNP A2B1 (Fig. 5A). Consistently, the confocal microscopy results revealed that exogenous NP was colocalized with endogenous hnRNP A2B1 in the MEF cells and exogenous hnRNP A2B1 in the cytoplasm in HEK293T cells (Fig. 5B and C). Moreover, CO-IP assays were performed to determine the direct relationship between hnRNP A2B1 and NP. We observed that NP could be pulled down by endogenous or exogenous hnRNP A2B1 in THP-1 and HEK293T cells, respectively (Fig. 5D and E). To further investigate which domain of hnRNP A2B1 is important for the interaction with NP, three mutants of hnRNP A2B1 were generated based on the functional domains of hnRNP A2B1 (Fig. 5F). Confocal microscopy data showed that those hnRNP A2B1 mutants were all remained in the nucleus when overexpressed in the HEK293T cells. Interestingly, co-expression of SFTSV NP and mutants RRM1 or RRM1-2 showed colocalization with NP and mutants in the cytoplasm, while the mutant RGG was still kept in the nucleus with NP distributing in the cytoplasm (Fig. 5G), suggesting that the RRM1 domain of hnRNP A2B1 was important for interaction with SFTSV NP in the cytoplasm. Furthermore, CO-IP results verified that exogenous NP and hnRNP A2B1 mutants RRM1 and RRM1-2 could be mutually pulled down in the HEK293T cells (Fig. 5H). These results suggest that SFTSV NP could mediate the retention of hnRNP A2B1 in the cytoplasm *via* interaction with the RRMs domain of A2B1 directly.

### HnRNP A2B1 interacts with SFTSV 5’ UTR

**Fig 6:**
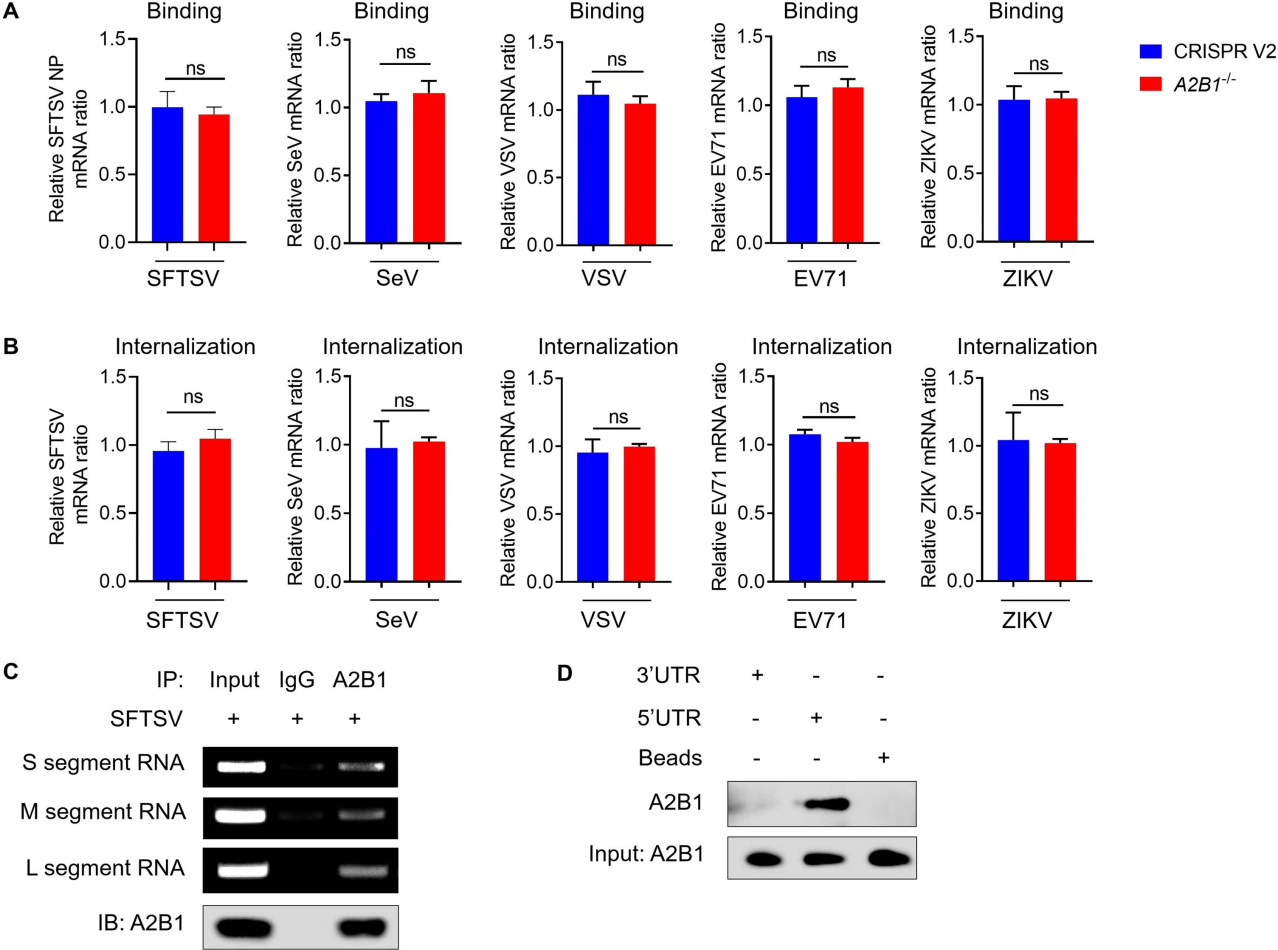
HnRNP A2B1 interacts with the 5′ UTR of SFTSV RNA. (**A**) WT and *A2B1*^-/-^ MEF cells were infected with SFTSV, SeV, VSV, EV71, and ZIKV at an MOI of 10 at 4°C for 1 h, *SFTSV NP*, SeV, VSV, EV71, and ZIKV mRNA levels were analyzed with RT-qPCR. (**B**) WT and *A2B1*^-/-^ MEF cells were infected with SFTSV, SeV, VSV, EV71, and ZIKV at an MOI of 10 at 4°C for 1 h, following incubation at 37°C for 2 h, *SFTSV NP*, SeV, VSV, EV71, and ZIKV mRNA levels were analyzed with RT-qPCR. (**C**) MEF cells were infected with SFTSV at an MOI of 10 for 24 h, and the interaction between S, M, or L segment RNA of SFTSV and hnRNP A2B1 was detected with RNA immunoprecipitation (RIP). (**D**) HEK293T cells were transfected with HA-hnRNP A2B1 plasmids for 24 h, and the interaction between 3′ or 5′ UTR of SFTSV S segment RNA and hnRNP A2B1 was detected with RNA pulldown assay. Data were obtained from three independent experiments (*n* = 3). *P < 0.05, **P < 0.01, ***P < 0.001, ****P < 0.0001.

We demonstrated that hnRNP A2B1 was utilized for SFTSV, SeV, VSV, EV71, and ZIKV replication. To detect which step of the SFTSV, SeV, VSV, EV71, and ZIKV life cycle is modulated by hnRNP A2B1, virus binding and internalization assays were performed. Our results showed that hnRNP A2B1 showed no effect on the binding and internalization of SFTSV, SeV, VSV, EV71, and ZIKV (Fig. 6A and B). Moreover, to detect whether hnRNP A2B1 participated in SFTSV replication *via* binding with viral RNA, cellular RNA was immunoprecipitated by hnRNP A2B1 during SFTSV infection. The L, M, and S segments of SFTSV RNA were then detected from immunoprecipitated products with PCR, and our results verified the interaction between hnRNP A2B1 and three SFTSV RNA segments (Fig. 6C). The untranslated regions (UTRs) are highly conserved and crucial for the replication, transcription, and packaging of the viral genome (30). Hence, the biotin-labeled 5′ and 3′ UTR of S segment RNA of SFTSV were synthesized to determine the interaction between UTR and hnRNP A2B1. Interestingly, we found that hnRNP A2B1 could interact with 5′ UTR but not 3′ UTR (Fig. 6D). These results suggest that the hnRNP A2B1 may enhance SFTSV replication by interacting with 5′ UTR of SFTSV RNA segments.

## DISCUSSION

SFTS, caused by SFTSV, is characterized by fever, thrombocytopenia, and multiple organ dysfunction, with a mortality rate of up to 30% (31). However, there are currently no specific treatments and the underlying pathogenic mechanism of SFTSV is poorly understood (32). Cellular proteins, especially those involved in replication and translation, are used by viruses to multiply within infected cells. A comprehensive understanding of virus-host factor interactions is critical to understanding viral pathogenesis. The hnRNP protein family is actively involved in the replication and maturation process of the virus (33).

It is known that hnRNP A2B1 is the RNA-binding protein that is associated with RNA splicing, metabolism, and transport in the nucleus (20). Here, we found that hnRNP A2B1 was unregulated in SFTSV-infected cells. hnRNP A2B1 was translocated from the nucleus to the cytoplasm during the infection of SFTSV and other RNA viruses, including SeV, VSV, EV71, and ZIKV. All these viruses replicate in the cytoplasm, utilizing host machinery for RNA synthesis and protein translation. Each virus requires host factors for efficient replication, including proteins involved in RNA stability, translation, and immune evasion. These viruses form specialized replication complexes that protect viral RNA from degradation and facilitate efficient replication. They all employ mechanisms to modulate host immune responses, including interferon signaling and other antiviral pathways (34, 35). Although all these are RNA viruses, their replication mechanisms are somewhat different. Negative-sense RNA viruses (SFTSV, VSV, and SeV) carry RNA genomes that must be transcribed into positive-sense RNA before translation can occur. This involves RdRp synthesizing mRNA and antigenomes (36). Positive-sense RNA Viruses (EV71 and ZIKV) have genomes that can be directly translated by host ribosomes into viral proteins. The viral RdRp synthesizes new RNA genomes from the positive-sense template (37). SFTSV has a segmented genome, which requires coordinated replication and packaging of multiple RNA segments (10). Non-segmented genomes (VSV, SeV, EV71, and ZIKV), simplifying the replication and packaging process. EV71 utilizes an internal ribosome entry site for initiating translation, independent of the cap structure usually required for eukaryotic mRNA translation (38). Cap-dependent translation (ZIKV, VSV, and SeV) relies on cap-dependent translation mechanisms (39).

After this RNA virus infection, hnRNP A2B1 can sense and undergo a translocation from the nucleus to the cytoplasm. Our results indicated translocation of the hnRNP A2B1 that was consistent with the translocation of other members of hnRNPs. The hnRNPs usually reside in the nucleus and can undergo the redistribution phenomenon under virus infection. It has been reported that hnRNP L is mainly localized in the nucleus in mock-infected cells but it is redistributed to the cytoplasm after being infected with Foot-and-Mouth Disease Virus (40). After EV71 infects host cells, hnRNP A1 was transferred from the nucleus to the cytoplasm (41). It is known that SFTSV replication occurs exclusively in the cytoplasm (42). In this study, we used co-immunoprecipitation to confirm and validate that the NP of SFTSV interacts with the hnRNP A2B1 in SFTSV-infected and NP-transfected cells. SFTSV NP can mediate the translocation of the hnRNP A2B1, and the RRM1 domain of the hnRNP A2B1 was important for interaction with NP and retention of the hnRNP A2B1 in the cytoplasm. Generally, hnRNP family proteins are known as nuclear proteins that contain nuclear localization signal (NLS) domains, like hnRNP H, hnRNP K, and hnRNP U (43–46), which are critical for the nuclear translocation process. Consistently, we found that the RRM1 domain of the hnRNP A2B1 showed colocalization with a nucleus, indicating the existence of the NLS domain, while SFTSV NP could promote the retention of the hnRNP A2B1 RRM1 domain in the cytoplasm. Thus, the hnRNP A2B1 could be retained in the cytoplasm by SFTSV NP *via* capturing the RRM1 domain during SFTSV infection.

Meanwhile, we also observed that the hnRNP A2B1-RGG which lacks an NLS domain entered the nucleus. We believe that the RGG (arginine-glycine-glycine) domain in hnRNP A2B1 is known for RNA binding and interactions with other proteins. This domain might mediate interactions with other nuclear import factors or nucleic acids that facilitate its nuclear localization (47). Proteins with RGG motifs can interact with import receptors other than importin-α/β (48). For example, transportin-1 can recognize glycine-rich sequences and mediate nuclear import (49). RNA-binding proteins, including hnRNP A2B1, often utilize their RNA-binding domains to facilitate nuclear import (50). The binding of hnRNP A2B1-RGG to specific RNA molecules might influence its nuclear localization, potentially utilizing RNA transport pathways. While the absence of a classical NLS in hnRNP A2B1-RGG poses a challenge for nuclear import, several alternative mechanisms can facilitate its entry into the nucleus. These include the piggyback mechanism, passive diffusion, alternative nuclear import pathways, and post-translational modifications (51). Interactions with other nuclear proteins, RNA, and import receptors play crucial roles in this process.

The hnRNP A2B1 itself was found important for SFTSV replication using the hnRNP A2B1 knockout and non-knockout cell lines. Although knocking out hnRNP A2B1 can have some effects on cellular homeostasis, temporarily inhibiting hnRNP A2B1 during critical phases of viral replication might reduce viral load while minimizing disruption to cellular RNA processes (52). The reversible inhibition would allow cellular functions to resume once the antiviral treatment is discontinued (53). The potentially harnessing compensatory mechanisms within the cell could help mitigate the effects of hnRNP A2B1 inhibition on cellular RNA homeostasis (54). For example, other RNA-binding proteins might partially compensate for the loss of hnRNP A2B1 function (55). Inhibiting the role of hnRNP A2B1 in viral replication offers a promising strategy for combating RNA virus infections. However, due to its essential functions in maintaining cellular RNA homeostasis, this approach must be carefully designed to avoid compromising normal cellular processes. Selective, temporal, and targeted inhibition, along with an understanding of compensatory cellular mechanisms, could help achieve this balance, leading to effective antiviral therapies with minimal side effects.

In addition, we found that the hnRNP A2B1 was not involved in the binding and internalization process of SFTSV, but interacted with 5′ UTR of SFTSV RNA tightly, indicating that the hnRNP A2B1 could promote SFTSV replication *via* interaction with SFTSV RNA. We also found the hnRNP A2B1 could promote RNA replication of other RNA viruses including SeV, VSV, EV71, and ZIKV. Actually, the proviral role of the hnRNP A2 has been reported in response to the infection of the Japanese encephalitis virus (JEV), which could interact with JEV proteins (core protein and nonstructural protein 5) and RNAs in the cytoplasm and promote the synthesis of JEV RNAs (56). Moreover, the hnRNP A2B1 was found to act as a positive regulator in viral RNA synthesis of influenza A virus by interaction with the viral NP (17). To some extent, our and others’ previous studies indicate that the role of the hnRNP A2B1 could be conserved and important for SFTSV or other RNA virus infections (17, 24, 26, 27). We hypothesized that the hnRNP A2B1 was recruited and retained by virus-related proteins in the cytoplasm and exploited as the virus replication platform for viral RNA replication and mRNA synthesis. Moreover, the hnRNP A2B1 plays different functions in RNA virus infection and DNA virus infection. The hnRNP A2B1 was discovered as a novel DNA recognition receptor that inhibits DNA virus HSV-1 replication in an IFN-dependent manner (26). In DNA virus infections, hnRNP A2B1 primarily operates in the nucleus, whereas in RNA virus infections, its role is more prominent in the cytoplasm. For DNA viruses, hnRNP A2B1 is heavily involved in splicing and processing of viral mRNAs. By contrast, for RNA viruses, its role leans more toward stabilizing viral RNAs and facilitating their translation. Both DNA and RNA viruses manipulate hnRNP A2B1 to alter host mRNA splicing and stability, but the specific interactions and outcomes can vary significantly depending on the type of virus (17, 24, 27, 51).

The effect of the hnRNPs as proviral or antiviral factors varies in different viruses (33). The hnRNPs play a crucial role in regulating viral replication in three ways. First, they bind directly with viral genomes to influence viral propagation. Previous studies have shown that hnRNP K interacts with HCV RNA to suppress HCV particle production (28), while hnRNP C interacts with the early 3′-untranslated region of human papillomavirus type 16 (HPV16) and activates HPV16 late mRNA splicing, contributing to HPV16’s pathogenicity (57). hnRNP A1 binds to the 5′ and 3′ ends of murine norovirus 1 and SARS Coronavirus viral RNA to help RNA circularization (58), while hnRNP Q interacts with the 3′ end of the transmissible gastroenteritis coronavirus genome to play a positive role in viral replication (59). Second, hnRNPs interact with viral-encoded proteins to regulate viral replication. hnRNP H interacts with the dengue virus non-structural one protein and helps the virus multiply (60). A previous study identified hnRNP H1 as interacting with HCV core protein to modulate HCV or cellular functions during HCV infection (61). Lastly, hnRNPs bind with both the viral genome and the viral-encoded protein to function. Previous studies have shown that hnRNP K and hnRNP A2B1 are virus-supportive factors interacting with hepatitis E virus RNA at promoter regions (29), while hnRNP K interacts with nonstructural proteins of Sindbis virus, enterovirus 71, hepatitis B virus, and viral subgenomic mRNA to facilitate viral replication (62–64). However, the specific influence of hnRNP A2B1 in the process of SFTSV infection has not been investigated, our identification of the mechanism of interaction of the hnRNP A2B1 with SFTSV NP will be important in understanding SFTSV infection.

The previous studies demonstrated that the hnRNP A2B1 played a role in some RNA viruses, with unknown mechanisms (17, 27, 33). In this study, we demonstrated that hnRNP A2B1 can upregulate the replication of all RNA viruses tested including SFTSV, VSV-GFP, SeV, EV71, and ZIKV. These results suggest that hnRNP A2B1 upregulation of viral replication is most likely conserved among RNA viruses. We demonstrated that hnRNP A2B1 was translocated from the nucleus to the cytoplasm under RNA virus infections, suggesting translocation of hnRNP A2B1 from the nucleus to the cytoplasm is crucial for RNA virus replications. We then used SFTSV as a model to demonstrate the mechanism of hnRNP A2B1 in the promotion of RNA virus replication. We found that overexpression of SFTSV NP can also cause hnRNP A2B1 translocation from the nucleus to the cytoplasm and that the SFTSV NP interacted with the RRM1 domain of A2B1. We further demonstrated that the hnRNP A2B1 interacted with the 5′ UTR of SFTSV RNA. In conclusion, we revealed that the hnRNP A2B1 upregulate viral RNA replication is conserved among RNA viruses; the mechanism of hnRNP A2B1 in promotion of SFTSV viral RNA replication is that SFTSV NP interacted with the hnRNP A2B1 to retain it in the cytoplasm where the hnRNP A2B1 interacted with the 5′ UTR of SFTSV RNA to promote the viral RNA replication. Our findings suggest that hnRNP A2B1 has the potential to be used as a drug target for anti-RNA virus therapy.

## MATERIALS AND METHODS

### Cells and viruses

Vero, MEF, and HEK293T cells were cultured in DMEM medium (Gibco, Beijing, China) containing 10% fetal bovine serum (FBS; Gibco, Auckland, New Zealand) and 1% streptomycin-penicillin (p/s) at 37°C with 5% CO_2_. THP-1 cells were maintained in RPMI 1640 medium (Gibco, Beijing, China) supplemented with 10% FBS (Gibco, Auckland, New Zealand), and induced with Phorbol 12-myristate 13-acetate (PMA, 100 ng/mL) for 48 h to promote cell differentiation. After treatment for 24 h, THP-1 cells were replaced with a new medium without PMA.

BMDM cells were isolated from mouse (6–10 weeks of age) hint legs, using RPMI 1640 medium to flush marrow from femurs into a 10 cm dish. Then add 10 ng/mL M-CSF (Macrophage Colony Stimulating Factor), which is an essential regulator of monocyte/macrophage proliferation to differentiation and survival.

SFTSV (strain JS2011-013-1) was cultivated in Vero cells at an MOI of 0.01, the culture medium was changed to DMEM containing 2% FBS after infection for 2 h and collected cellular supernatant 6 to 7 days post-infection, freezing and thawing twice for cell disruption totally, stored at −80°C.

### CRISPR/Cas 9 system

*A2B1*^-/-^ THP-1 and MEF cell lines were generated using the CRISPR/Cas9 system, and the gene-specific single-guide RNA (sgRNA) sequence was designed by the online CRISPR Design Tools (). The sgRNA sequences were as follows: 5′-CACCGGTTCCTCAAACTTTCTTCTG-3′ for human A2B1, and 5′-CACCGGGAATGGGGCCTTGCAGCCA-3′ for mouse A2B1. In brief, after LentiCRISPRv2-A2B1 or LentiCRISPRv2-Ctrl, pMD2.G, and psPAX2 were packaged together with polyetherimide and co-transfected into HEK293T for 48–60 h. The supernatant was collected for precipitation with PEG8000, and the resuspended lysate was mixed with THP-1 cells previously containing 5 g/mL polybrene. The selected clonal cells were identified by gene sequencing and Western blot.

### Antibodies

Primary antibodies specific for A2B1 and β-actin were obtained from Santa Cruz Biotechnology (Dallas, TX). Primary antibodies specific for Lamin A, Rab5, LaminB1, Akt, and IgG were obtained from Cell Signaling Technology (Beverly, MA). Primary antibodies specific for Flag-tag and HA-tag were obtained from Abbkine (Wuhan, China). Anti-DNA antibody was obtained from Merck Millipore (Darmstadt, Germany). Antibodies specific to SFTSV NP were maintained in our laboratory.

### Mice and *in vivo* virus challenge

Animal care and uses were adhered to the Guide for the Care and Use of Laboratory Animals of the Chinese Association for Laboratory Animal Science, with the approval of the ethics committee of the Medical School, Wuhan University (2019YF2013). The *A2B1*^fl/f^ and conditional *A2B1*^fl/fl^*Lyz2-Cre^–/–^* mice on a C57BL/6J background were kindly provided by Professor Xue-tao Cao from Nankai University in Tianjin, China. The *A2B1* gene was identified with PCR. Mice were bred in pathogen-free conditions. Age- and sex-matched mice (6–8 weeks old) were challenged with SFTSV with 1 × 10^8^ plaque-forming units (PFU) by intraperitoneal injection. For analyzing SFTSV replication, mice were sacrificed 2 days post-infection.

### Western blot analysis

For cell samples, the cell lysate was diluted with RIPA lysis buffer (Beyotime, Shanghai, China), briefly ultrasonicated, and stored at −80°C. For tissues, about 0.2 g mouse spleen were homogenized in RIPA lysis buffer containing 1% PMSF with an electric homogenizer for 3 min, centrifuged at 12,000 *g* at 4°C for 15 min, briefly ultrasonicated, and stored at −80°C. All the protein samples were heated at 95°C for 10 min and separated by 12% SDS-PAGE, transferred to polyvinylidene difluoride membranes (Millipore, Burlington, Massachusetts, USA) blocked with 5% non-fat milk Tris-buffered saline and Tween 20 for 1 h, and incubated with corresponding primary antibodies overnight at 4°C, followed by incubation with secondary antibodies for 1 h and washed with phosphate-buffered saline with Tween 20. The protein bands were detected using the ChemiDoc touch imaging system (Bio-Rad, Hercules, California, USA) and processed using ImageLab software.

### RNA extraction and RT-qPCR

RNA was isolated using TRIzol Reagent (Invitrogen, Carlsbad, CA). For tissues, 0.1 g of tissues was homogenized in 1 mL trizol. For cell samples, 2 × 10^6^ cells were lysed in 0.5 mL trizol. The lysate was added with 0.5 mL chloroform, shaken thoroughly, incubated for 3 min, and centrifuged for 10 min at 12,000 *g* at 4°C, the supernatants were added with 0.5 mL isopropanol, centrifuged with the same condition after incubating for 10 min. The RNA was washed with 75% ethanol and the total RNA was dried in air.

The cDNA was synthesized using the High Capacity cDNA Reverse Transcription Kit (Invitrogen, Carlsbad, CA). RT-qPCR was performed with specific primers listed in Table S1. Relative mRNA concentrations were calculated with the 2^−ΔΔCt^ method, normalizing with β-actin.

### Nuclear and cytoplasmic protein extraction

Cytoplasmic and nuclear proteins were extracted using a nuclear and cytoplasmic protein extraction kit (Solarbio, Beijing, China) according to the manufacturer’s instructions. The equal amounts of cytoplasmic and nuclear proteins from the cell lysates were determined with a Western blot. Lamin A and Lamin B1 were used for internal reference in the nucleus, and Rab5 and Akt were used for cytoplasm.

### Confocal microscopy

MEF or THP-1 cells were infected with SFTSV (MOI = 10) for 48 h. MEF and HEK293T cells were transfected with indicated plasmids for 24 h. The cell samples were fixed in 4% (wt/vol) paraformaldehyde for 20 min, permeabilized with 0.2% Triton X-100, blocked in 5% bovine serum albumin (BSA) for 30 min, incubated with corresponding primary antibodies overnight at 4°C, and labeled with fluorescently secondary antibodies for 1 h. Nuclei were stained with 4′,6-diamidino-2-phenylindole (DAPI; Beyotime, Shanghai, China) for 15 min. The cell samples were observed using a Leica sp8 confocal laser microscope with a 63× objective lens. Images were processed using Leica Application Suite X software.

### Coimmunoprecipitation

To confirm the interaction indicated proteins, cells were infected with SFTSV for 48 h or transfected with appropriate plasmids for 24 h for co-immunoprecipitation (CO-IP). The cells were lysed with IP cell lysis buffer (Beyotime, China), incubated with specific antibody or IgG as a negative control overnight at 4°C. Protein A + G agarose (Beyotime, Shanghai, China) was added and gently rotated at 4°C for 3 h. The mixture was then centrifuged and washed five times with PBS. The beads were collected and resuspended with SDS-PAGE loading buffer, and analyzed with Western blot.

### RNA immunoprecipitation (RIP) assay

RNA immunoprecipitation was performed using the RNA Immunoprecipitation Kit (BersinBio, Guangzhou, China) according to the manufacturer’s instructions. MEF cells (2 × 10^7^) were infected with SFTSV at an MOI of 10 for 24 h, the cell lysate was incubated overnight at 4°C with magnetic protein A/G beads conjugated with antibody of hnRNP A2B1 or IgG as negative control, the immunoprecipitated A2B1 and RNA was extracted and analyzed with Western blot and PCR, respectively.

### RNA pulldown assay

RNA pulldown assay was performed using the RNA pulldown Kit (BersinBio, Guangzhou, China) according to the manufacturer’s instructions. HEK293T cells (2 × 10^7^) were transfected with HA-A2B1 plasmids for 24 h, cytoplasmic proteins were extracted, and incubated with biotin-labeled RNA probes to form RNA-protein complexes. The streptavidin-labeled magnetic beads were used to separate from the other components of the incubation solution. After elution, the proteins that interacted with specific RNA were detected with Western blot.

### Statistical analysis

All the calculations and graphs were compared and made by Student’s *t*-test or analysis of one-way ANOVA with GraphPad Prism Software, and at least three independent experiments were performed in triplicate. All the data indicate accepted the level of statistical significance of *P* < 0.05.
